# Cathinone Use Disorder in the Context of Slam Practice: New Pharmacological and Clinical Challenges

**DOI:** 10.3389/fpsyt.2020.00705

**Published:** 2020-07-22

**Authors:** Benoit Schreck, Marylène Guerlais, Edouard Laforgue, Célia Bichon, Marie Grall-Bronnec, Caroline Victorri-Vigneau

**Affiliations:** ^1^ Department of Addictology and Psychiatry, CHU Nantes, Nantes, France; ^2^ French Institute of Health and Medical Research, Universités de Nantes et de Tours, INSERM UMR 1246 SPHERE, Nantes, France; ^3^ Department of Clinical Pharmacology, CHU Nantes, Nantes, France

**Keywords:** slam, slamming, chemsex, party and play, new psychoactive substances, cathinones, cathinone use disorder

## Abstract

**Background:**

“Slam” has emerged since 2008 as a new international phenomenon among men who have sex with men (MSM); it consists of the intravenous injection of drugs before or during planned sexual activity. The practice of slam is associated with the use of psychostimulants, including synthetic cathinones.

**Methods:**

All spontaneous notifications (Nots) of slam practice reported between January 2012 and October 2019 at the Nantes addictovigilance center in France were collected and analyzed. The purpose of this work was to analyze cases of slam to characterize cathinone use disorder according to the diagnostic criteria of the Diagnostic and Statistical Manual of Mental Disorders, Fifth Edition (DSM-5) and to further our knowledge of slam practice based on data on drug use, risk taking and harmful consequences.

**Results:**

We collected 39 slam Nots. The severity of cathinone use disorder was mild, moderate and severe for 18%, 12%, and 58% of the patients, respectively. “Much time spent using cathinone” was the diagnostic criterion most often cited (82%).

**Conclusions:**

To the best of our knowledge, our study is the first to evaluate the presence of a cathinone use disorder. Cathinone use disorder seems particularly important in this population of users, and negative consequences of slam practice appear quickly.

## Introduction

Slam has emerged as a new phenomenon in Western Europe (1, 2) among men who have sex with men (MSM) since 2008 and has also emerged in Southeast Asia (3–5), North America (6, 7), and Australia (8, 9), where it is called “party and play” or “intensive sex partying”. According to the French Monitoring Centre for Drugs and Drug Addiction (OFDT), slam is a form of chemsex defined by (i) the use of psychostimulants (ii) through the intravenous route of administration (iii) in a sexual context (10). For several years, there has been emerging evidence focusing on high-risk sexual practices and infectious harms (11–15) but not on the substances used in this specific context. In Europe, and more particularly in France, this practice is associated with the use of specific psychostimulant substances in the synthetic cathinone family. Synthetic cathinones are a large group of stimulants chemically related to cathinone, a phenethylamine that is the most potent amphetamine-like compound naturally present in the khat plant (*Catha edulis*, Peter Forsskål, 1775) (16). The molecular structure of cathinone can be modified in various ways to produce a series of compounds chemically related to it, and this technique has been used to circumvent international drug laws (17). The pharmacological properties of synthetic cathinones differ from molecule to molecule, but they exert their effects by increasing the concentration of catecholamines such as dopamine, serotonin, and norepinephrine in the synaptic cleft (16). Animal studies have shown that synthetic cathinones have reinforcing properties and abuse liability (18). Synthetic cathinones are used for their stimulant and entactogenic effects; desired effects include euphoria, intensification of sensory perceptions, increase in sociability, increase in energy, and increase in sexual performance, which is precisely why they are used in slam (16). In fact, Weatherburn et al. (19) identified motivations for combining sex and these drugs; they increase libido, confidence, disinhibition, and stamina, enhance qualities of the sex, and make other men seem more attractive, increase physical sensations, intensify perceptions of intimacy, and facilitate a sense of sexual adventure. Nevertheless, cathinone use is not without risks, since fatalities have been associated with the consumption of various cathinones (20). Mephedrone, 3-methylmethcathinone (3-MMC), and 4-methylethcathinone (4-MEC) are the most commonly substances used in slam (10, 21), but new synthetic cathinones appear monthly on the illicit drug market, making knowledge of these substances and their impact on users even more complex. In 2018, 130 different cathinones were identified by the European Monitoring Centre for Drugs and Drug Addiction (21). No accurate data are available in the literature about the specific effects of synthetic cathinones in the context of slam, how they are used, and if they induce substance use disorder, negative consequences, withdrawal syndrome, tolerance, or craving.

France is the only European country with a national system dedicated to observing and assessing the abuse and dependence potential of psychoactive substances, medicines and drugs (22). The addictovigilance centers are responsible for the collection of cases of drug dependence, abuse, and misuse related to the taking of psychoactive substances through notifications (Nots) by health professionals. These centers are organized in a network, and there are thirteen centers spread throughout France. The missions of the addictovigilance centers are defined in the public health code (23). The three major ones are (i) to collect data and evaluate the dependence potential of the identified psychoactive drugs; French regulations make it mandatory to report all cases of serious abuse and drug dependence related to the intake of psychoactive substances or herbs as well as any other drug or product (24, 25); (ii) to provide information on the risk of abuse or dependence on psychoactive substances; and (iii) to carry out scientific research.

Since 2012, the addictovigilance center of Nantes has been notified of several cases of the use of psychoactive substances, including synthetic cathinones, in the context of slam practice. It is therefore within the framework of its missions that the Nantes addictovigilance center has been able to obtain data on the slam practice in the Pays de la Loire region.

The purpose of the present study was to analyze cases of slam reported to the Nantes addictovigilance center to characterize cathinone use disorder and to further our knowledge of slam practice based on data on drug use, risk taking, and harmful consequences.

## Materials and Methods

### Those Who Notify

The Pays de la Loire addictovigilance center is located in the Nantes University Hospital. Notifications of psychoactive substance use in a sexual context come from general practitioners, specialists working in hospitals or in private offices, and other health professionals. The AIDES association, which is particularly involved in prevention and risk reduction, also participates in Nots. In fact, AIDES works with people with sexually transmitted infections (STIs) [including human immunodeficiency virus (HIV) and hepatitis C virus (HCV)] and aims to reduce risky practices. All healthcare professionals (regardless of their field of expertise) are required to anonymously report cases of serious drug abuse and dependence associated with the use of substances or plants with psychoactive effects (26, 27). These spontaneous Not reports by healthcare professionals are key for determining “real life” drug misuse and abuse and for identifying new nonmedicinal drugs that present a risk to public health.

### The Collected Data

The addictovigilance center in Nantes has sensitized practitioners concerned with slam practice to collect, in the course of notification, important data related to drug risk evaluation in this particular context. According to the French National Agency for Medicines and Health Products Safety (ANSM) notification form, the three types of information collected regarded sociodemographic characteristics, medical history, and drug use; drug use was defined according to the substance disorder diagnostic criteria of the DSM-IV (28) for the Nots before 2013 and the criteria of the DSM-5 (29) for all other Nots. In this context of the analysis of mandatory Nots, which is one of the main duties of the French addictovigilance centers, no permission to use a database was required (23).

Nots collected at the addictovigilance center of Nantes between January 2012 and October 2019 were extracted using the keyword “slam” in the center’s database. The data were as follows:

- Sociodemographic characteristics (age, family status, employment status);
- Medical history (HIV status, HCV status, lifetime and current substance use disorder, lifetime, and current psychiatric disorder);
- Information related to drug use (drug name and family drug name, dose by intake and frequency of administration, routes of administration, effects sought, effects felt, unpleasant symptoms felt after taking drug, perceived negative consequences on health and in the socioprofessional environment, tolerance, weaning, craving, desire to stop, and risk taking related to use).

The data were collected and analyzed with Microsoft® Excel software.

## Results

### Characteristics of the Nots

**Figure 1:**
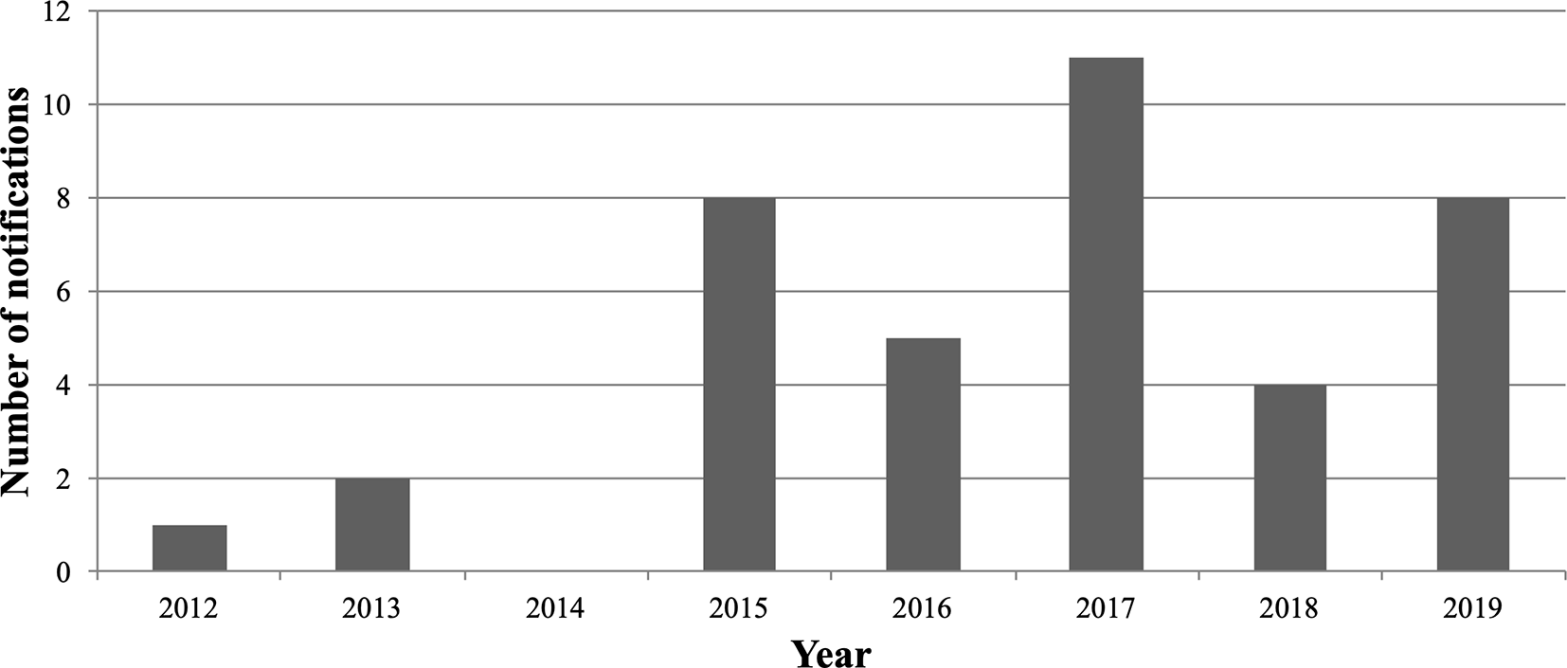
Number of notifications to Nantes addictovigilance center per year.

In total, the Nantes addictovigilance center collected 39 slam Nots. The first notification was reported at the Nantes addictovigilance center in 2012. The number of reports varied across the years, but we observed the maximum number of cases in 2017. The results are shown in **Figure 1**.

### Characteristics of the Subjects

We found that 34 slammers were the subject of 39 Nots corresponding to different slam sequences. Over the 2012–2019 period, two slammers were the subject of two Nots, and one slammer was the subject of four Nots. Sociodemographic and medical history data are shown in **Table 1**. The median age was 38 years (min–max: 23–63), and the median duration of slam practice was 3 years (1–5). More than four-fifths of the sample were HIV positive (82%, 18/22), 27% (6/22) were infected with HCV, and 18% (4/22) were coinfected with HIV and HCV. More than two-thirds of the subjects (69%, 22/32) reported a history of substance use disorder.

**Table 1:** Sociodemographic and medical history of the sample.

	N	Median (Min–Max) Percent (N)
Age (years)	34	38 (23–63)
Duration of slam practice (years)	30	3 (1–5)
Marital status: couple	29	62% (18)
Employment	29	62% (18)
Substance use disorder	32	69% (22)
Mood disorder	22	18% (4)
HIV-positive	22	82% (18)
HCV-positive	22	27% (6)
Syphilis	22	27% (6)

### Characteristics of the Slam Practice

#### Psychoactive Substances

**Figure 2:**
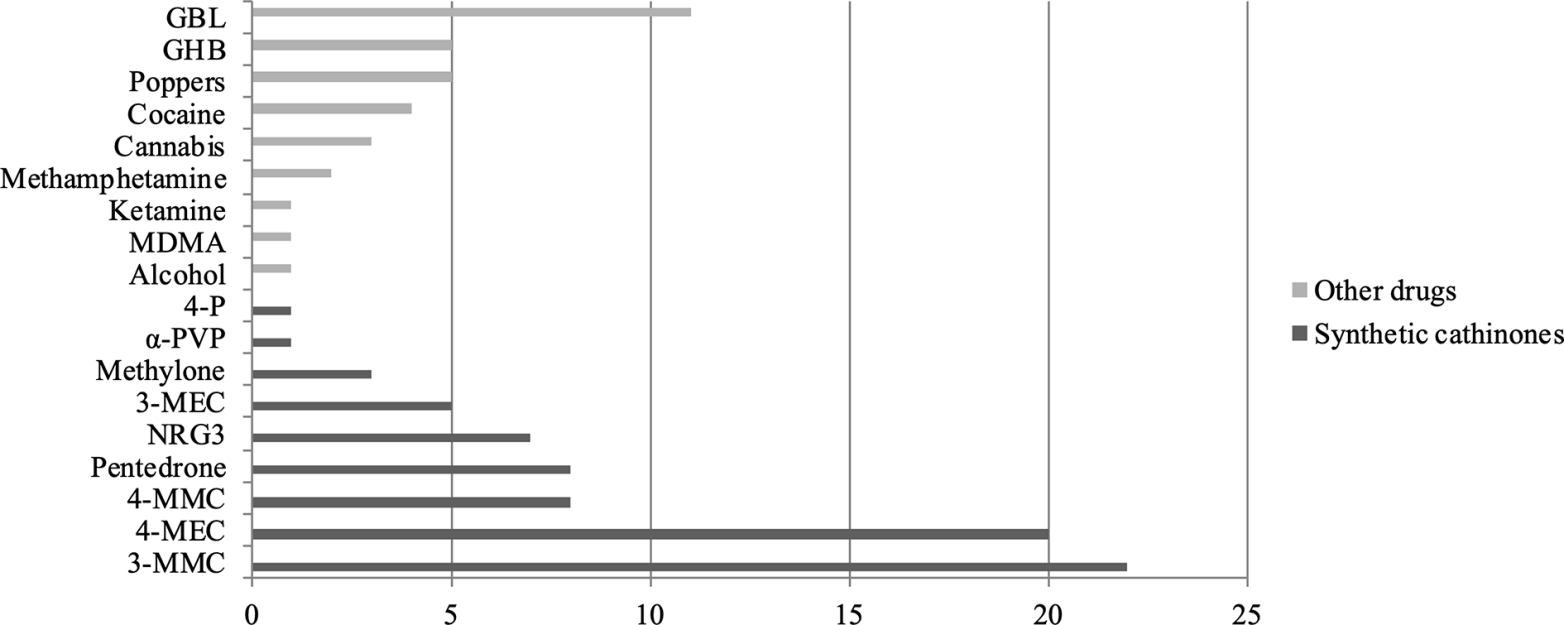
Number of psychoactive substances used.

During slam sessions, all the subjects used drugs in the cathinone family: 4-MEC (20/34 users), 3-MMC (22), mephedrone or 4-methylmethcathinone (4-MMC) (8), pentedrone (8), NRG3 (a combination of cathinones) (7), 3-methylethcathinone (3-MEC) (5), methylone (3), α-pyrrolidinovalerophenone (α-PVP) (1), and 4-P (1). Some slammers used cathinones in combination with other psychoactive substances; among the 18 drugs used during slam sessions, nine substances did not belong to the cathinone family: γ-butyrolactone GBL (11), γ-hydroxybutyrate (GHB) (5), cocaine (4), poppers (5), cannabis (3), methamphetamine (2), methyl​enedioxy​methamphetamine (MDMA) (1), ketamine (1), and alcohol (1). The set of psychoactive substances used according to the number of citations is shown in **Figure 2**.

#### Routes of Administration and Frequency

For all users, the administration of cathinones was intravenous. The frequency of the intake varied according to each user, ranging from two per session to one administration every hour, until the exhaustion of the availability of the drugs. The frequency of slam practice was monthly for 29% (10/34 users) of the users, multimonthly for 21% (7) of the users, weekly for 12% (11), and multiweekly for 6% (2); this information had not been entered for four slammers.

#### Polydrug Use

The median number of drugs used when practicing slam was 3 (1–9). Polydrug use occurred in 85% (29/34) of the subjects; nevertheless, we did not have the information to say that the polydrug use took place during the same session or during different sessions. However, the users reported never mixing drugs within a syringe.

### Characteristics of Cathinone Use

#### Cathinone Use Disorder Diagnosis

Only one Not was reported in 2012, before the publication of the DSM-5. The slammer described in this Not presented with cathinone dependence according to the DSM-IV (28). From 2013, 29 slammers out of 33 presented with cathinone use disorder according to the DSM-5 criteria (29). The severity of cathinone use disorder was mild, moderate and severe for 18%, 12%, and 58% of the patients, respectively. The median number of diagnostic criteria met was 6 (0–11).

#### Cathinone Use in the Context of Slam

**Figure 3:**
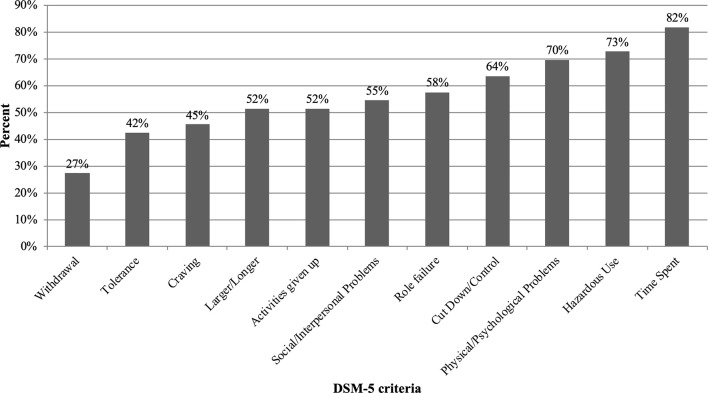
Percentage of 33 slammers meeting the DSM-5 substance use disorder criteria.

The percentage of the 33 slammers presenting with each DSM-5 Substance Use Disorder criterion is shown in **Figure 3**. “*Much time spent using cathinone*” was the diagnostic criterion most often cited (82%). Then, the “*hazardous use*” criterion (“*recurrent substance use in situations in which it is physically hazardous*”) and the “*physical/psychological problems*” criterion (“*substance use is continued despite knowledge of having a persistent or recurrent physical or psychological problem that is likely to have been caused or exacerbated by the substance*”) were cited in 73% and 70% of the cases, respectively. The criteria on craving, tolerance and withdrawal, at 45%, 42%, and 27%, respectively, were less present among the slammers.

Health consequences (physical or psychological), with the exception of those experienced during the “comedown” period, and social consequences were entered in the Nots for some users and are shown in **Table 2**. Unpleasant symptoms were reported by the users in the “comedown” phase in the days following the use of the psychoactive substances in the sexual session. Several users described intense asthenia (24%, 8/34), anxiety with sadness (24%, 8/34), hallucinations (9%, 3/34), depersonalization (6%, 2/34), and paresthesia (6%, 2/34).

**Table 2:** Health and social consequences of cathinone use in the context of slam practice.

*Health consequences*	65% (22/34)
*Psychological*	26% (9/34)
Sadness	56% (5/9)
Asthenia	33% (3/9)
Anxiety	33% (3/9)
Loss of self-confidence	33% (3/9)
Apathy	22% (2/9)
Amnesia	11% (1/9)
Sleep disorder	11% (1/9)
Hallucinations	11% (1/9)
Suicidal ideation	11% (1/9)
Loss of appetite	11% (1/9)
*Physical*	38% (13/34)
Pain	31% (4/13)
Injection site injury	23% (3/13)
Syphilis infection	15% (2/13)
Anal lesion	8% (1/13)
Tachycardia	15% (2/13)
Weight loss	8% (1/13)
Sweating	15% (2/13)
Urinary retention	8% (1/13)
Tremor	8% (1/13)
Low blood pressure	8% (1/13)
HCV infection	8% (1/13)
HIV infection	8% (1/13)
*Social consequences*	53% (18/34)
Separation	44% (15/34)
Loss of work	24% (8/34)

The desired effects reported by the subjects during the administration of synthetic cathinones were the following:

- Letting go: “well-being, relaxation, hovering” (62%, 21/34);
- Stimulation: “sexual arousal, increased endurance, increased alertness” (50%, 17/34);
- Disinhibition (35%, 12/34);
- Anesthesia and analgesia: “performing hard sexual practices, decreasing the sensation of pain” (18%, 6/34);
- Increased sensations (12%, 4/34);

### Evolution of Drug Use

Among the 39 Nots, five involved the same three users over the 2012–2018 period, which allowed us to identify an evolution in terms of drug use. For the first slammer, Nots were reported in 2015, 2017, 2018, and 2019. In 2015, this subject reported using mephedrone, GBL, and poppers during slam sessions. In 2017, he reported using 3-MMC, pentedrone, and α-PVP. In 2018 and 2019, he reported only using 3-MMC. There was also a change in drug use patterns: in 2015, he began to use monthly by the intravenous and intranasal routes. Then, the route of administration became exclusively intravenous and he began using weekly in 2017 and 2018. He tried several times to cut down or stop participating in the slam practice but quickly relapsed; nevertheless, the Nots of 2019 informed us about a decrease in frequency with a multimonthly slam practice. For the second slammer whose Nots were reported across an 8-month interval in 2017 and 2018, there was no notable difference in the substances used and the modalities of use during the slam sessions. For the third slammer, two Nots were reported at a 1-month interval in 2016. He reported using cathinones multiweekly in a slam context. Strong cravings were reported in both Nots.

## Discussion

### Comparison With National Cases and International Literature

Batisse et al. (30) synthesized all cases of chemsex practice reported to the French addictovigilance network for the period from 2008 to 2017. This analysis concerned chemsex practice without specifying the proportion of slam. We observed similarities between the national analysis and our regional slam analysis regarding (i) age, (ii) frequency of HIV, HCV and other STIs, (iii) psychiatric history, and (iv) substances most commonly consumed (3-MMC and 4-MEC). Polydrug use was high in both groups but was higher among the regional cases (national cases: 64%, regional cases: 85%), which might be related to the systematic inquiry in these cases, resulting from the regional awareness of the professionals involved and therefore the specific selection of slam cases. In contrast, differences emerged between the national chemsex analysis and the regional slam analysis. A history of substance use disorder was clearly more prevalent in our sample (national cases: 16%, regional cases: 69%); this difference may be explained by the systematic nature of the Nots inquiry in our sample. Finally, the mean duration of the practice was on average more than 1 year in the national cases and more than two and a half years (m=2.7, sd=±1.5) in the regional cases.

Thus, our cases appeared to be similar to those identified in the rest of France, except regarding drug use, which appeared to be more severe in this regional sample (substance use disorder and polydrug use background). This difference can be explained by the more complete data obtained in our sample relative to those in the routine spontaneous Nots usually performed in addictovigilance centers, i.e., those analyzed in Batisse et al. (30).

The median age of the slammers was similar to those found in international articles (4, 8, 31, 32). We also found that the slammers tended to have a job, which has been previously reported (8). The frequency of HIV infection has been very variable in the literature, ranging from 0.6% (33) to 100% (34), but our sample of slammers was in the upper range (4, 8, 15, 31, 33–37). In contrast, HCV infection was less common in our sample than in the literature (8, 15, 31, 35–38). There were similar findings regarding STIs (38). According to the international literature, slammers mostly use methamphetamine (8, 31, 34, 37), a psychostimulant substance about which we have much pharmacological data (39). Our entire sample reported using synthetic cathinones, about which we have very little data regarding their effects and toxicities (40, 41). Methamphetamine is known for its effects on sexual activity (42–44) through the activation of neurons in brain regions of the mesolimbic system that are involved in the regulation of sexual behavior (43, 44). This drug provides sexual disinhibition, endurance and sexual arousal (4, 31, 32),which is probably why it is used in slam. Methamphetamine is widely available in the United States of America (6), the United Kingdom (21), and Australia (31) but is almost absent in France or sold only in Paris at very high prices, explaining the preferential use of cathinones in the practice of slam in our sample. Considering the extremely high percentage of cathinone use disorder in our sample (88%), we can assume that the synthetic cathinones used by slammers seem as addictogenic as methamphetamine, which is a very potent amphetamine derivative (39). As in our sample of slammers, the literature has also noted the strong representation of poppers and GHB–GBL use in the context of slam practice (8). Risk behaviors associated with substance use, such as sharing of syringes or small equipment, were present at a higher level in our sample of slammers than in the literature, where they seldom occurred in more than 10% of slammers (31, 33, 34). The motivations of slammers to practice slam were the same as those found in the article of Bui et al. (31), that is, seeking disinhibition, endurance, sexual stimulation, and increased sensations.

Finally, the regional sample also seemed to have the same characteristics as those found in the international literature in terms of risk of STIs and motivations for slam practice. Nevertheless, the substances used were not the same, and risk behaviors associated with substance use were more severe in our sample.

### Cathinone Use Disorder

Our study on slamming explored the synthetic cathinone use and related disorder. To the best of our knowledge, our study is the first to address the existence of a cathinone use disorder, both among slammers and the general population. This is an aspect of slam practice that is crucial to explore, considering that we have demonstrated that a cathinone use disorder was screened among 88% of the slammers; this result highlights the highly addictive nature of synthetic cathinone when used in this specific context. We did not find any articles describing cathinone use disorder. Nevertheless, it is interesting to take a closer look at khat, a plant that contains mainly cathinone, and although khat use disorder is not still recognized as a diagnostic entity, disorders related to khat use are being more frequently described in the literature. In fact, Duresso et al. (45) reported a prevalence of 73.8% of “khat use disorder” in a sample of 400 frequent Ethiopian chewers aged 16 and above. In a 2019 systematic review by Mihretu et al. (46), the prevalence of problematic khat use ranged from 20.6% to 80.7% in khat-chewing communities in Ethiopia, Saudi Arabia, and Somalia. Thus, it is clear that khat, which contains a derivative of amphetamine, can induce substance use disorder and that by their chemical proximity, synthetic cathinones are potentially capable of inducing substance use disorder. Cathinone use disorder seems to be an actual diagnostic entity that has not been clearly explored; therefore, our data are unlike previously published data, especially as they relate to a specific population with a particular practice.

In our sample, the three most prevalent criteria for cathinone use disorder were the time spent, hazardous use, and physical/psychological problems. The criteria on physical dependence (tolerance and withdrawal) were the two least represented criteria in our sample. As previously described by many authors and initially by Goodman (47), the key symptoms of addiction are the loss of control and the continuation of the use despite the negative consequences. We demonstrated that is also the case for cathinone use disorder. This is consistent with the international literature regarding khat use. For example, in the article by Duresso et al. (45), the criterion “continuation use despite physical/psychological problems” was the most prevalent; “tolerance” and “withdrawal” were among the least represented. The systematic review by Mihretu et al. highlighted the psychological dependence that complicates the use of khat (46). The results regarding stimulant use disorder are more nuanced. The most represented diagnostic criteria among methamphetamine and cocaine users were “withdrawal”, “much time spent” and “social/interpersonal problems” according to Gilder et al. (48), and “quit/control”, “hazardous use”, and “social/interpersonal problems” according to Saha et al. (49).

Furthermore, the very high prevalence of cathinone use disorder is even more noteworthy when we consider the low duration of the slam practice in our sample. We can see how the use of cathinones can quickly become invasive in all aspects of life, with harmful consequences and loss of control. The presence of cathinone use disorder seemed obvious and impacted the majority of slammers in our sample, but it is necessary to consider the polydrug use and sexual context playing a large role in the practice of slam. Indeed, almost all slammers declared polydrug use during slam sessions, which can disrupt their perception of cathinone’s effects or reinforce the addictive nature of cathinones, leading to overreporting a cathinone use disorder. Thus, polydrug use can make it difficult to analyze and understand substance use in the context of slamming.

### Pharmacological and Toxicological Aspects

Some data are available on the pharmacodynamics, pharmacokinetics, and toxicological properties of synthetic cathinones; these are studies using animals (40, 50, 51) and humans (52, 53), like clinical trials (54, 55). Moreover, we have seen that polydrug use affects the majority of subjects (85%). Polydrug use is more the rule than the exception in slam practice, and the use of combinations of substances can lead to pharmacodynamic and pharmacokinetic interactions. For example, the combination of synthetic cathinones, substances with high adrenergic potency, with sildenafil (phosphodiesterase type 5 inhibitor) and a vasodilator (such as poppers) would increase cardiovascular risk. Therefore, practicing slam exposes individuals to a high risk of pharmacodynamic interactions as well as pharmacokinetic interactions. Indeed, some antiretrovirals used in the treatment of HIV (such as ritonavir) are powerful enzymatic inhibitors likely to increase the toxicity of synthetic cathinones (56, 57); as a reminder, 82% of the users in our sample were infected with HIV. Thus, polydrug use in slam leads to increased physical (mainly cardiovascular) and neuropsychiatric risks, especially if the user suffers from preexisting illnesses. Moreover, high and frequent doses of drugs are often being used in long sessions of slam (4, 8, 31, 32, 58, 59), which increases the risk of toxicity of each product. The Nots did not provide the frequency and the number of injections and therefore the total quantity of drugs administered per session. Another important element concerns the drugs used as reported by the slammers. We have no certainty that what they used actually corresponded to what they had bought. Since 1999, in France, the National Detection System of Drugs and Toxic Substances (SINTES) scheme has intended to document the toxicological composition of illegal substances in circulation in France. The information incorporated in this system comes from two sources: the submission to the OFDT of the toxicology test results performed on seizures by law enforcement laboratories (French National Forensic Science Institute, Forensic Sciences Institute of the French gendarmerie and Customs laboratories) and investigations conducted by the OFDT on samples of substances obtained directly from users. Collection of these data is governed by a strict regulatory framework and conducted by specifically trained survey workers. The analysis of substances by SINTES makes it possible to know the scams being used, which are of three types: different products, unexpected quantities, and adulterations by cutting agents. In 2018, synthetic cathinones were the third most scammed products (60); this was particularly true with 3-MMC, which has been the subject of a large number of substitutions, especially ephylone. Moreover, at the European level (21), we know that a large proportion of the new psychoactive substances come from the Internet drug market, and a 2016 European Monitoring Centre for Drugs and Drug Addiction (EMCDDA) report (61) mentioned frequent scams on websites. Because of these scams, the risks of intoxication are high; depending on the particular molecule, there can be extreme variations in the doses that cause an effect as well as the toxicity thresholds. Thus, slam practice, beyond the risks of infection and risks related to sexual acts, poses a real pharmacological danger.

### Strengths and Weaknesses

Our study has some limitations. First, health professionals and workers primarily report the most severe cases, which could have led to a selection bias. Moreover, our sample size was also relatively small, which limits the representativeness of this population of slammers; however, we must be careful not to stigmatize slammers (4, 15), as many users do not have problematic behavior and do not seek help from health professionals. In addition, the Nantes area differs from other areas in terms of its sexual networks and patterns of drug use, and it may not be possible to extrapolate our findings to other geographical areas. We assume that concerns about the disclosure of sensitive issues, such as sexual behavior and drug use, might still persist and that socially desirable answers could have been given to health professionals. Furthermore, this was a cross-sectional study that makes it very difficult to measure changes in the practices of slam users. Unfortunately, the notification form of the ANSM does not capture information relevant to behavioral addictions because it was designed to contribute to a pharmacological database. However, slamming is a complex behavior because it involves both substance use and sexual activity at the same time, with the drug use being at the service of the sexual behavior; sex and drugs are strongly entangled. Psychostimulant drugs make sexual practice more intense, more compulsive, and less controllable (4, 32, 59). These elements are also strongly suggestive of hypersexuality or sex addiction in slammers, but there are no data in the international literature regarding sex addiction in slammers (or chemsexers), probably because it can be difficult to distinguish between substance use disorder and sex addiction. In fact, we can assume that sex addiction could be a confounding factor in the detection of a cathinone use disorder in our sample; the desire to cut down, control, or stop the behavior, the craving, the persistence of behavior despite psychic, physical, or social consequences can also be connected to sex addiction. Unfortunately, the sexual aspect has not been explored in the Nots because this notification form was made to highlight new psychoactive substances, new patterns of use behavior or new types of harm related to substance use and was not made to characterize sex addiction or a hypersexuality disorder.

However, these limitations are compensated for by the strengths of the study. Our results are original, and there is no similar published work in the literature that provides a detailed analysis of intravenous substance use in a sexual context. All Nots were made by health professionals or workers in the health sector who had accurate data on each patient in their care; we have exhaustive data on substances used in the practice of slam, and screening for a substance use disorder was done according to the DSM-5 benchmarks.

### Future Directions

Our work contributes to a better understanding of slam practice. We have seen that substance use disorder seems particularly important in this population of users, and negative consequences of slam practice quickly appear. This work partially addresses the lack of pharmacological knowledge on cathinone and associated substances that are usually used in slam; the goal is to better understand the effects of substances used in the slam practice to develop effective medical management and risk reduction counseling. Future work should focus particularly on the issues of hypersexuality/sex addiction, motivations, repercussions, and precipitating and maintaining factors in these practices among slammers. It is essential to maintain vigilance regarding slam practice, to try to analyze user trajectories in the evolution of their practice and not to stigmatize this community but rather to develop future relevant prevention messages.

## Data Availability Statement

The raw data supporting the conclusions of this article will be made available by the authors, without undue reservation.

## Ethics Statement

Ethical review and approval was not required for the study on human participants in accordance with the local legislation and institutional requirements. Written informed consent for participation was not required for this study in accordance with the national legislation and the institutional requirements.

## Author Contributions

BS, MG-B, CV-V, CB, EL, and MG collected the data. BS, MG, and CB performed the analysis. BS, MG-B, and CV-V wrote the paper. MG and CB helped to draft the manuscript and participated in the discussion. All authors contributed to the article and approved the submitted version.

## Conflict of Interest

The authors declare that the research was conducted in the absence of any commercial or financial relationships that could be construed as a potential conflict of interest.
